# A Cost-Effectiveness Tool for Informing Policies on Zika Virus Control

**DOI:** 10.1371/journal.pntd.0004743

**Published:** 2016-05-20

**Authors:** Jorge A. Alfaro-Murillo, Alyssa S. Parpia, Meagan C. Fitzpatrick, Jules A. Tamagnan, Jan Medlock, Martial L. Ndeffo-Mbah, Durland Fish, María L. Ávila-Agüero, Rodrigo Marín, Albert I. Ko, Alison P. Galvani

**Affiliations:** 1 Center for Infectious Disease Modeling and Analysis, Yale School of Public Health, Yale University, New Haven, Connecticut, United States of America; 2 Department of Epidemiology of Microbial Diseases, Yale School of Public Health, Yale University, New Haven, Connecticut, United States of America; 3 Department of Biomedical Sciences, College of Veterinary Medicine, Oregon State University, Corvallis, Oregon, United States of America; 4 Pediatric Infectious Diseases Department, Hospital Nacional de Niños “Dr. Carlos Sáenz Herrera”, San José, Costa Rica; 5 Programa de Control de Vectores, Ministerio de Salud, San José, Costa Rica; 6 Centro de Pesquisas Gonçalo Moniz, Fundação Oswaldo Cruz, Ministério da Saúde, Salvador, Bahia, Brasil; 7 Department of Ecology and Evolutionary Biology, Yale University, New Haven, Connecticut, United States of America; University of Oklahoma Health Sciences Center, UNITED STATES

## Abstract

**Background:**

As Zika virus continues to spread, decisions regarding resource allocations to control the outbreak underscore the need for a tool to weigh policies according to their cost and the health burden they could avert. For example, to combat the current Zika outbreak the US President requested the allocation of $1.8 billion from Congress in February 2016.

**Methodology/Principal Findings:**

Illustrated through an interactive tool, we evaluated how the number of Zika cases averted, the period during pregnancy in which Zika infection poses a risk of microcephaly, and probabilities of microcephaly and Guillain-Barré Syndrome (GBS) impact the cost at which an intervention is cost-effective. From Northeast Brazilian microcephaly incidence data, we estimated the probability of microcephaly in infants born to Zika-infected women (0.49% to 2.10%). We also estimated the probability of GBS arising from Zika infections in Brazil (0.02% to 0.06%) and Colombia (0.08%). We calculated that each microcephaly and GBS case incurs the loss of 29.95 DALYs and 1.25 DALYs per case, as well as direct medical costs for Latin America and the Caribbean of $91,102 and $28,818, respectively. We demonstrated the utility of our cost-effectiveness tool with examples evaluating funding commitments by Costa Rica and Brazil, the US presidential proposal, and the novel approach of genetically modified mosquitoes. Our analyses indicate that the commitments and the proposal are likely to be cost-effective, whereas the cost-effectiveness of genetically modified mosquitoes depends on the country of implementation.

**Conclusions/Significance:**

Current estimates from our tool suggest that the health burden from microcephaly and GBS warrants substantial expenditures focused on Zika virus control. Our results justify the funding committed in Costa Rica and Brazil and many aspects of the budget outlined in the US president’s proposal. As data continue to be collected, new parameter estimates can be customized in real-time within our user-friendly tool to provide updated estimates on cost-effectiveness of interventions and inform policy decisions in country-specific settings.

## Author Summary

Using data on Zika virus, microcephaly, and Guillain-Barré syndrome cases from Brazil and Colombia, we compute ranges for the probability of a microcephaly outcome in infants born to Zika-infected women (0.49% to 2.10%, based on data from Northeast Brazil) and the probability of Guillain-Barré syndrome in Zika-infected individuals (0.02% to 0.06% in Brazil and 0.08% in Colombia). These results have allowed us to create a web-based cost-effectiveness tool that quantifies the relationship between the cost of an intervention and the number of Zika virus cases, as well as loss of disability-adjusted life years, that it can avert. Our tool thus identifies the threshold at which a given intervention, such as vector control, may be deemed cost-effective or very cost-effective in a variety of settings according to WHO criteria, in terms of the Zika burden that could be averted and the cost of such an intervention.

## Introduction

In April 2015, the first confirmed case of mosquito-borne Zika virus in the Americas was reported in Brazil [1]. Since then, the virus has spread to 41 countries and territories across the Americas, Oceania, the Pacific Islands, and Africa [2], with over 1.5 million suspected and confirmed cases [1]. In the US, sexually transmitted or travel associated cases have been reported in 40 States and the District of Columbia. Furthermore, transmission has been reported in the Commonwealth of Puerto Rico, the Virgin Islands of the US, and the Territory of American Samoa [3]. There are projections of millions more cases in both the countries Zika has already reached and others within which it has yet to emerge [1], including predictions of local transmission in the Gulf Coast of the US [4]. There is strong scientific consensus that Zika virus can cause Guillain–Barré syndrome (GBS) [5] and that a Zika infection in pregnant women can cause microcephaly in their infants [1,6,7], vision-threatening ocular lesions [8], *in utero* growth restriction, fetal deaths, stillbirths, and central nervous system lesions [9]. On February 1, 2016, the World Health Organization (WHO) declared the outbreak an International Health Emergency [10]. Without vaccines or medication to treat Zika infections, vector control remains the only immediate option to combat this outbreak.

However, there are hesitations regarding whether extensive efforts likely necessary to contain Zika, such as intensive scale-up of vector control, the application of insecticides, or the implementation of new technologies that could include genetically modified or *Wolbachia*-infected mosquitoes, would be worth the substantial costs, logistical challenges, and potential environmental repercussions [11]. For example, genetically engineered male *Aedes aegypti*, the offspring of whom die prior to maturity, are being piloted against the ongoing Zika outbreak. This approach is relatively expensive for middle income countries, requiring $1.9 million in the first year and $384,000 each year thereafter for an urban population of 50,000 [12].

The investment in Zika control should be considered relative to the disease burden that could be averted and the resources available in the country. For example, the Brazil National Development Bank has allocated $136.6 million [13] towards combating mosquito-borne diseases. The Costa Rican Department of Social Security has committed $745,724 for community-led elimination of breeding sites, through removal of containers of stagnant water in high-risk regions, and the Ministry of Health is planning on allocating $373,712 exclusively to the control of Zika. Considering the populations in Brazil and Costa Rica, these investments represent only about $0.66 and $0.23 per citizen, respectively. Moreover, while Brazil and Costa Rica have similar per capita income, the same investment would be valued differently in countries with different resource constraints. Cost-effectiveness analysis provides a framework under which such investments can be studied for each particular case. As with any global pandemic, international effort to control the outbreak should be led by agencies such as the WHO and by countries with the resources and expertise necessary to confront the threat. To combat the current Zika outbreak, the US President requested from Congress in February the allocation of $1.8 billion [14]. Of this total, $250 million has been allocated to the Commonwealth of Puerto Rico for the prevention of Zika infection in pregnant women and for medical costs associated with Zika. To curtail the Zika outbreak internationally, the United States Agency for International Development would receive $335 million and the State Department $41 million to address Zika in Latin America and the Caribbean. The remaining requested funds would be directed towards outbreak management in the US, expanded vector control measures, and vaccine development. Fundamental to these investment decisions is the quantification of the costs, including any environmental risks, of an intervention balanced by its value in terms of the health burden that it would likely avert. We offer quantitative insight into the health burden that an unchecked Zika epidemic might incur and provide an interactive web application (http://zika.cidma.us/) that can be employed by health authorities to evaluate the cost-effectiveness of programs under consideration for Zika control.

This study aims to evaluate the cost-effectiveness of expenditures towards Zika control intervention, based on available data. To support these calculations, we also estimated the probability of microcephaly arising from a Zika-infected pregnancy, parameterized with case data from Northeast Brazil, and the probability of GBS following a Zika infection, using Brazilian and Colombian case data. We further estimated the number and burden of cases of Zika-linked microcephaly and GBS expected to occur across the Americas in 2016, should the Zika epidemic remain unabated. We also provide thresholds of price and effectiveness combinations for interventions that would satisfy WHO criteria for cost-effectiveness. Additionally, our interactive web tool has the flexibility to be updated as more information becomes available on this emerging disease.

## Methods

### Microcephaly

We estimated the probability of a microcephaly case given a Zika infection during the first trimester of pregnancy, as the proportion of the Zika-related microcephaly births among all births to mothers infected with Zika during the first trimester of pregnancy [15]. Our web tool also has the flexibility to adjust the duration of risk during pregnancy, as reports emerge suggesting that the risk could extend beyond early pregnancy.

Birth counts are totaled for the outbreak in Northeast Brazil, where Zika was first confirmed to have reached Latin America in mid-April 2015 [1, 16] and where cases have already begun to decline [17]. Given our assumption that only first-trimester Zika infections can cause microcephaly, we expect Zika-liked microcephaly to begin arising around October 15, 2015, six months after the first infection. Cumulative case data on suspected and confirmed microcephaly cases were obtained for Northeast Brazil until April 2, 2016, with the first report containing cumulative case counts from November 15, 2015 [17]. To forecast the microcephaly cases yet to arise, we applied a linear regression over the weekly reported cases for 2016 in Northeast Brazil [17].

Excess microcephaly cases, above what would be expected for Northeast Brazil during this time period, were assumed to be linked to Zika infection. This excess is calculated as the difference between the estimated total microcephaly cases during the outbreak in Northeast Brazil and the expected non-Zika related microcephaly births for the same duration and region. To estimate the total microcephaly births for the Northeast Brazil outbreak, we took into account the reporting sensitivity, i.e., the proportion of confirmed cases from among all investigated microcephaly cases in the region [17] and the expected total reported microcephaly births. The estimated total reported microcephaly births is then the sum of our forecast of newly reported microcephaly cases for the remaining outbreak in Northeast Brazil and the latest available reported microcephaly cases for Northeast Brazil (5,380 as of April 2, 2016).

The expected microcephaly cases attributable to causes other than Zika were based on prevalence estimates of microcephaly prior to the outbreak from Brazil (0.5 per 10,000 births) [18] and the highest reported prevalence from the US (12 per 10,000 births) [19] to account for possible underreporting before the outbreak. These prevalence values were multiplied by expected births during the Zika-related microcephaly outbreak in Northeast Brazil to estimate expected microcephaly from other causes. The expected births during the outbreak in Northeast Brazil were estimated from the fraction of the Brazilian population in Northeast Brazil (28%) [20], the Brazilian population [21], and the Brazilian birth rate (14.931 per 1000 population) [22].

Since the attack rate in Northeast Brazil is unknown, to estimate the births by mothers infected in their first trimester, we used attack rates ranging from 23.5% (reported for the latest outbreak in Puerto Rico of chikungunya [23], a related arbovirus transmitted by the same mosquito species) to 77% (upper bound for Zika outbreak in Yap Island, Micronesia in 2007 [24]). The 2013–2014 French Polynesia Zika outbreak had an attack rate between those two bounds (66% [95%CI: 62–70] [15]). The supplement provides a table of the parameters used and their sources, as well as a flow diagram of the equations underlying our calculations of the probability of microcephaly given a Zika infection during the first trimester of pregnancy (S1 Table and S1 Fig).

The probability of microcephaly given Zika infection, in the absence of interventions targeted towards pregnant women, is estimated by multiplying the probability of microcephaly given a Zika infection in the first trimester of pregnancy, the geographic-specific birth rate, and the risk period divided by 365 days.

### Guillain-Barré Syndrome (GBS)

We estimated the probability of developing GBS given a Zika infection as the proportion of Zika-related GBS cases from among all Zika infections. Parameterization of our calculations are based on data from a 10-week period in Colombia (45,314 Zika cases and 231 GBS cases, from January 9th to March 19, 2016 [1,25]), adjusting for estimates suggesting that 82% of Zika infections are asymptomatic [24], and on the average annual GBS incidence for Colombia from 2008 to 2014 (242) [26]. Similarly, we recalculated the probability of developing GBS using the WHO estimate of 269 Zika-related GBS cases in Brazil [1] using the estimated epidemic size ranging from 443,502 to 1,301,140 for Brazil [27]. The lower and upper limits of this estimate are based on suspected cases of dengue fever for which dengue was ultimately excluded and on the attack rate of Zika virus during the 2013 French Polynesia Zika outbreak, respectively [27].

### Health and Economic Outcomes

The health impact of microcephaly and GBS was calculated in disability-adjusted life-years (DALY). We estimated the health impact of a single case of microcephaly in present-value terms (discounting at 3% annually), assuming a 79.7% probability of survival through the first year of life [28], an optimistic life expectancy of 35 years given survival beyond that year, and the 0.16 disability weight assigned to living with severe intellectual disability [29]. In the absence of available medical costs for microcephaly, we conservatively used the lifetime direct medical cost associated with a case of mental retardation, estimated as $179,760 in lifetime expenses for the US [30, 31]. In estimating the health impact of a single case of GBS, we conservatively assumed a 5% probability of death [1], a 9% probability of severe motor impairment (0.402 disability score) for 6 months, and an 84% probability of moderate generalized musculoskeletal problems (0.344 disability score) for three weeks [29, 32]. The average age of a case in the Colombian outbreak was 43 years [1], and we assumed this was also the average age of death (if death occurred). The direct medical cost per case of GBS was $56,863 for the US [30, 33].

The medical costs for both microcephaly and GBS were updated to 2015 USD using the Consumer Price Index Inflation Calculator [30], and then converted to location-specific costs for each country or region using the World Bank purchasing power indices for medical expenses [34]. Our base case estimates for the costs associated with microcephaly and GBS are highly conservative in that they do not incorporate reduced productivity and quality of life, as well as other indirect costs associated with the conditions, such as educational and support services for microcephaly. To conduct analyses that account for these costs, our interactive tool allows the user to vary the per case cost associated with microcephaly and GBS.

We estimated the number of microcephaly and GBS cases averted as the product of Zika infections averted and the probabilities of microcephaly or GBS per Zika infection, respectively. The health burden was estimated as the product of the cases averted and the DALYs lost per case, for both microcephaly and GBS. Similarly, the economic burden was estimated as the product of the cases averted and the direct medical expense for each condition.

### Cost-Effectiveness Analysis

A net health benefit framework [35] combines health outcomes (here, in DALYs), economic costs, and a willingness-to-pay for DALYs to establish the value of an intervention in a particular setting. The net health benefit of a particular strategy is calculated as the DALYs it averts minus its net cost as a proportion of the willingness to pay threshold. The WHO has established two willingness-to-pay thresholds at the country level: the per-capita GDP or three times the per-capita GDP for interventions to be considered “very cost-effective” or “cost-effective,” respectively [36]. A positive net health benefit calculation at these willingness-to-pay thresholds indicates that the intervention fulfills the criteria for cost-effectiveness. This analysis is conducted from a government perspective, given our focus on the cost of intervention and direct healthcare costs.

### Interventions

We applied the net health benefits framework to both WHO thresholds at which investment would be deemed cost-effective and very cost-effective, respectively, to evaluate:

1. The economic cost that would be justified by an intervention which averted 10,000,000 Zika infections across Latin America and the Caribbean, corresponding to 1.6% of the population, compared to the $376 million expenditure proposed to be allocated by the US for Zika-related foreign aid;
2. The economic cost which would justify preventing 90% of the infections in pregnant women at risk in Puerto Rico;
3. The number of Zika infections that must be averted in Costa Rica to justify the $745,724 investment in community-led efforts to eliminate breeding sites;
4. The number of Zika infections that must be averted in Costa Rica to justify its $373,712 investment dedicated to the control of Zika;
5. The number of Zika infections which must be averted in Brazil to justify the $136.6 million investment in mosquito-borne disease control by the Brazilian National Bank;
6. The cost viability of disseminating genetically engineered mosquitoes for three years in a city of 50,000 inhabitants in different country-specific settings.

### Interactive Web Tool

Our interactive web tool was coded in Python (www.python.org) using the NumPy package for scientific computing (http://www.numpy.org/) and the Bokeh interactive visualization library (http://bokeh.pydata.org/).

## Results

**Fig 1:**
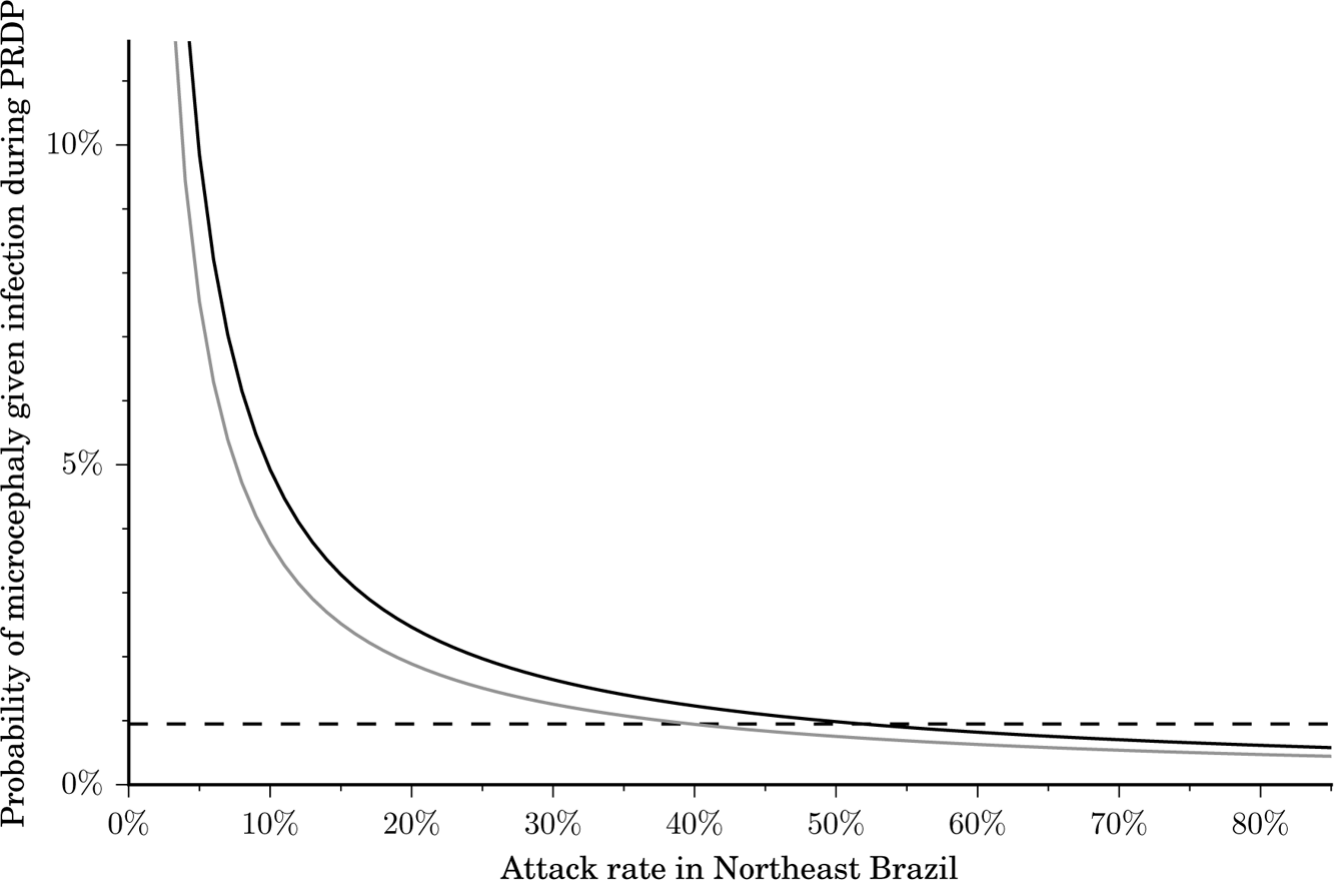
Probability of microcephaly in an infant given a Zika infection in the period of risk during pregnancy (PRDP). The probability is highly sensitive to the attack rate of Zika virus in Northeast Brazil, but not very sensitive to the expected microcephaly incidence for reasons other than Zika infection (black, 0.5 cases per 10,000 births; grey, conservative baseline of 12 cases per 10,000 births). Attack rates between 40% and 60% are compatible with the probability obtained in the 2013 French Polynesia outbreak (horizontal dashed line).

**Fig 2:**
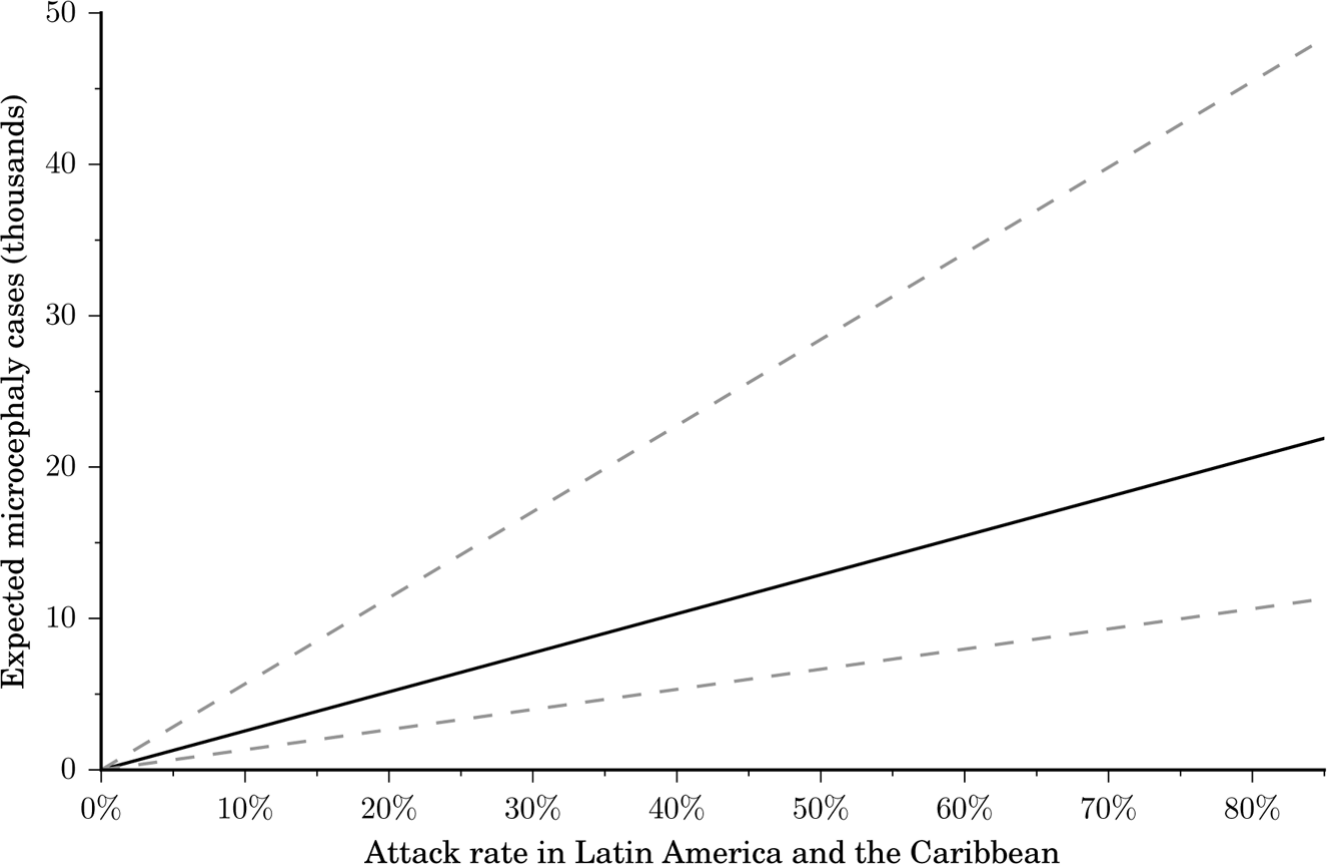
Expected microcephaly cases for Latin America and the Caribbean. The expected microcephaly cases depend on the attack rate, the birth rate, the population size, the non-Zika related microcephaly incidence, and the probability of microcephaly given a Zika infection in the first trimester of pregnancy. We used the probability of microcephaly observed for the French Polynesia 2013–2014 outbreak (0.95%, solid line) as the baseline for our calculations [15]. Our low and high estimates for the probability of microcephaly in Northeast Brazil (0.49% and 2.10%, dashed lines) encompass the estimate for the French Polynesian outbreak.

We estimated that an additional 94 microcephaly cases are likely to occur in Northeast Brazil between the last available report on April 2, 2016 through the end of April, after which the microcephaly outbreak is projected to dwindle in this region. We estimated the probability of a microcephaly case given a Zika infection during the first trimester to range between 0.49% and 2.10%. This probability is highly sensitive to the final attack rate for Zika in Northeast Brazil (Fig 1). Since the reported probability of 0.95% for the recent outbreak in French Polynesia [15] falls within our estimated range, we used this value in our base case parameter set. In our most conservative and less conservative scenarios we used 0.49% and 2.10%, respectively. We also estimated the probability of Zika-related GBS. Specifically, from the Brazilian data, we calculated that the probability of GBS given a Zika infection ranged from 0.02% to 0.06%, consistent with a recent estimate of 0.024% for the French Polynesia outbreak [5]. From the Colombian data our probability estimate was 0.08%. We used 0.06% in our base case parameter set, and 0.02% and 0.08% for our most conservative and less conservative scenarios, respectively. The final number of microcephaly and GBS cases predicted to occur throughout Latin America and the Caribbean depends on the attack rate for the region (Fig 2). For example, an attack rate of 5% would lead to a predicted 665 to 2843 microcephaly cases, 6,474 to 25,494 GBS cases, and a loss of 43,717 to 108,951 DALYs, whereas an attack rate of 40% would elevate those predictions to between 5,320 and 22,743 microcephaly cases, 51,790 to 203,951 GBS cases, and a loss of 349,738 to 871,610 DALYs. To put this potential health burden in context, the total dengue fever annual health burden is estimated as 89,500 DALYs in Latin America and the Caribbean [37].

**Fig 3:**
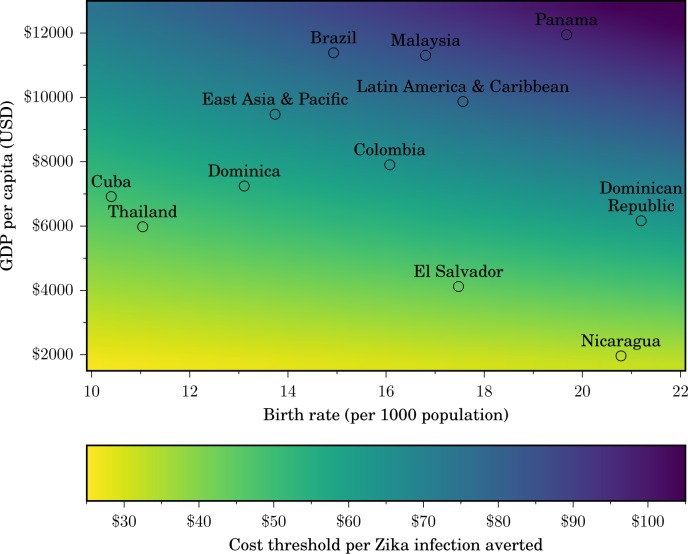
Cost-effective expenditure to avert a Zika infection. The maximum investment that would be cost-effective for a country to avert a Zika infection increases with the gross domestic product (GDP) per capita and with the birth rate. Some countries and regions at risk for Zika are indicated for illustration. A color scale indicates the cost-effectiveness threshold (from yellow: lowest cost; to purple: highest cost).

We estimated that each microcephaly case conservatively represents the loss of 29.95 DALYs and a direct medical cost of $91,102 per lifetime. Similarly, for GBS we estimated a health burden of 1.25 DALYs per case, as well as a direct medical cost per case of $28,818. The total DALYs likely to be averted by an intervention in a specific setting is influenced by demography (birth rate and population size), and the criteria for cost-effectiveness depend on the per-capita GDP (Fig 3). For example, our base case analysis suggests that an intervention that prevents 10,000,000 Zika infections across Latin America and the Caribbean, corresponding to 1.6% of the population, would be cost-effective for expenditures below $802 million and very cost-effective below $409 million, using the average regional per capita GDP and birth rate. Preventing infections in only 1.6% of the population is likely achievable since previous attack rates of Zika virus in Yap Island and French Polynesia have been estimated to range between 62% and 77% [15,24]. Thus, the $376 million for foreign aid in Latin America and the Caribbean proposed by the US President to combat Zika would likely be a very cost-effective investment.

These estimations conservatively assumed that the infections averted would be uniformly distributed among the entire population. Interventions that focus on preventing infection among pregnant women would be cost-effective for greater expenditure. We calculated that 13,490 pregnant women are at risk of Zika infection if the outbreak is unabated in Puerto Rico, assuming the same attack rate as French Polynesia and the estimated duration of the microcephaly outbreak for Northeast Brazil. If an intervention is able to avert 90% of those infections in pregnant women, it would be cost-effective at $195.4 million. This calculation suggests that the expenditure of $250 million for Puerto Rico is justified, given that the funding is allocated not only to the prevention of infection in pregnant women, but also medical costs associated with Zika cases.

Conversely, using the same framework we can also calculate the minimal number of Zika infections that an intervention with a fixed cost would have to avert to be deemed cost-effective. For example, the $745,724 that the Costa Rican Department of Social Security has allocated for community-led elimination of breeding sites would be cost-effective if it averted 8,321 Zika infections. The campaign would be considered very cost-effective if it averted at least 14,386 Zika infections. The additional $373,712 investment that the Costa Rican Ministry of Health plans to dedicate to the control of Zika needs to avert at least 4,170 Zika infections in the country to be considered cost-effective and at least 7,210 to be considered very cost-effective. In Brazil, the $136.6 million that its National Development Bank has committed, if dedicated exclusively to prevent Zika, would be cost-effective if it averts 1,640,934 infections and highly cost-effective if it averts 3,245,553 infections. These analyses are conservative given that the same mosquito vector also transmits dengue, yellow fever and chikungunya.

The cost-effectiveness of releasing genetically modified male mosquitoes whose offspring die before reaching adulthood depends on the expected Zika attack rate, the effectiveness of prevention, and the country in which the intervention is implemented. For example, in a Panamanian city with a population of 50,000 (e.g. Santiago de Veraguas) we estimated that the three-year implementation of this technology was cost-effective if it prevented 27,356 infections. The same intervention would not be not cost-effective in similarly populous cities located in countries with lower per-capita GDP (e.g., El Salvador or Nicaragua) or with lower birth rates (e.g., Cuba or Thailand), because the number of infections that must be prevented within the city would be greater than the entire population of the city.

**Fig 4:**
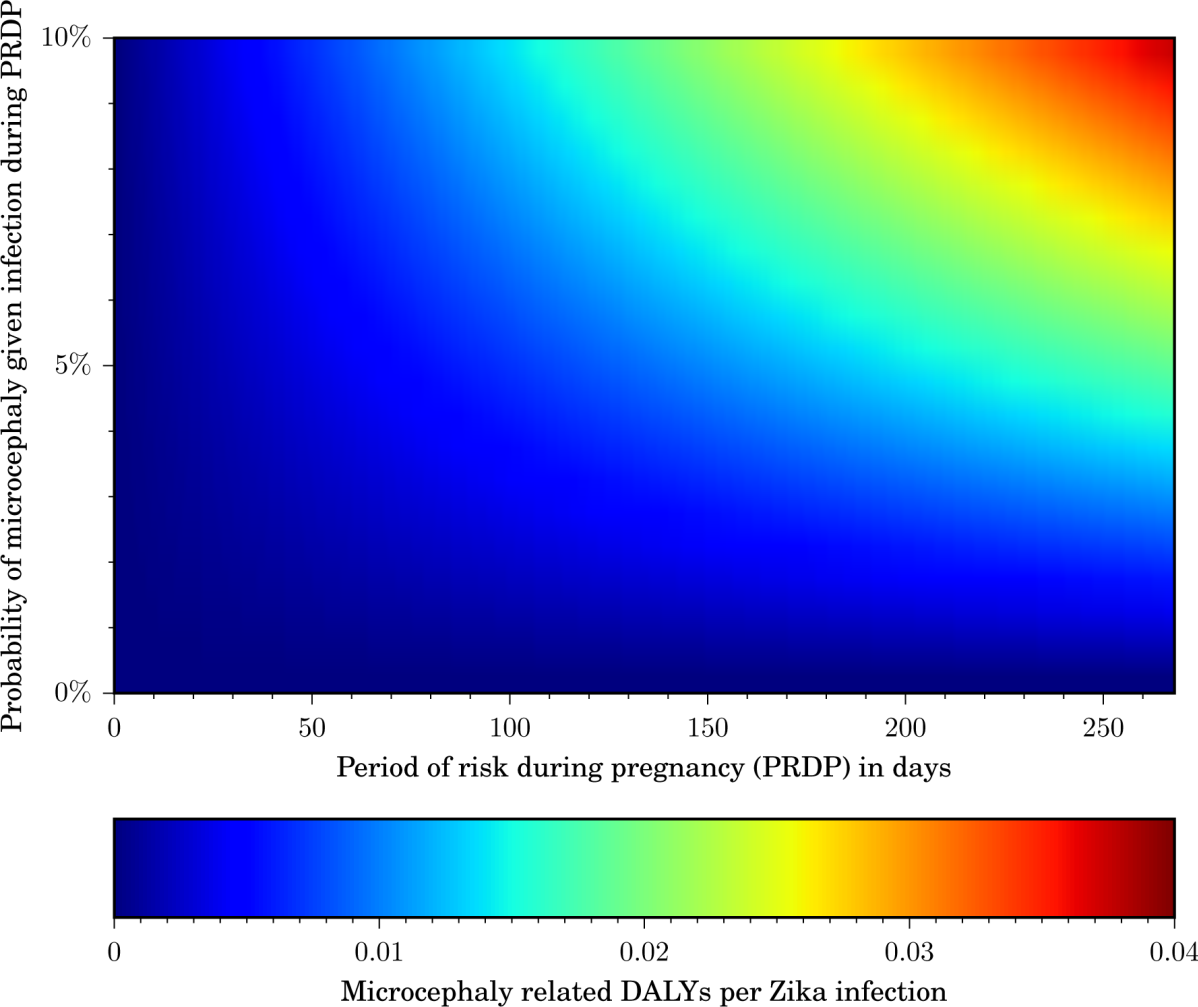
Average microcephaly-related DALYs per Zika infection. The DALYs lost per Zika infection increase with the period of risk during pregnancy (PRDP), as well as with the probability that a Zika infection during that period causes microcephaly. A color scale indicates the microcephaly-related DALYs lost per Zika infection (from blue: fewest DALYs per infection; to red: most DALYs per Zika infection).

We developed an interactive web tool for the evaluation of interventions beyond those illustrated here (http://zika.cidma.us/). Our tool allows policy makers to compute the cost-effective expenditure for an intervention that prevents a given number of Zika infections, or the number of infections which must be averted to justify an intervention cost. The user can vary the country or region of interest, which automatically adjusts the GDP, the population size, and the birth rate to generate setting-specific thresholds. Two key parameters that impact the DALY burden are the period of gestation during which a fetus would be at risk of Zika infection if the mother is infected, as well as the probability that a Zika infection of a pregnant woman within that period leads to microcephaly (Fig 4). The user can interactively modify those two parameters, as well as the probability of a GBS case given a Zika infection. For the analysis of programs with an emphasis on pregnant women, the user can specify a percentage of intervention effort directed specifically to pregnant women. To incorporate indirect medical costs and other expenses, our tool allows the user to specify the per-case cost of microcephaly and GBS. The output of our tool includes the combinations of cost and Zika infections averted for which intervention expenditure would be deemed as very cost-effective, cost-effective, or neither. Hover text displays exact values for the intervention cost, DALYs averted, and net health benefit. As information arises, our web-based tool can be adjusted to provide real-time projections of the health burden of the Zika outbreak in different countries.

## Discussion

Using data-driven analyses, we estimated the health and economic burden of an unchecked Zika epidemic and evaluate the conditions under which proposed interventions would be cost-effective. To parameterize our analyses, we calculated the probability that Zika infection leads to microcephaly or GBS, respectively. We developed a web tool to assist policymakers in their assessment of country-specific control measures.

Our results provide conservative estimation both of the burden of Zika, and of the expenditure justified for an intervention. Congenital Zika infection has been associated with a number of conditions beyond microcephaly, including vision-threatening ocular lesions [8], *in utero* growth restriction, fetal death, stillbirth, and central nervous system lesions [6, 9]. Additionally, given the severity of Zika-related cases of microcephaly [38], the life expectancy of 35 years is likely highly conservative. If the average life expectancy for these microcephaly cases is lower, interventions aimed at curtailing the ongoing Zika outbreak will avert a greater number of DALYs, since each year of life lost has a higher burden than a year lived with disability. As more data become available, the health burden associated with a single Zika infection should be updated.

For the results presented here, we did not consider the indirect costs related to GBS or microcephaly. Our tool allows the user to adjust the per-case cost for microcephaly and GBS, giving the user the option to include indirect costs if or when they are available for a specific country. Indirect costs can be important to assessments of cost-effectiveness from the societal perspective, particularly given that the economic losses for caregivers of children with microcephaly may be substantial. For example, in Puerto Rico we estimated total direct medical costs for microcephaly and GBS of $104 million in our base case and from $39 million in our most conservative scenario up to $159 million in our less conservative scenario. However, upon inclusion of both direct non-medical costs and productivity losses [30, 31, 33], the values rise to $736 million in the base case, with $280 million in the most conservative scenario and as high as $1.13 billion in the less conservative scenario. Even these estimates do not include other indirect costs such as specialized child-care support, parental productivity losses, or psychological toll of families with children with microcephaly, which are all substantial, yet difficult to quantify.

Our analyses are further conservative in their exclusion of the impact on other diseases that could be achieved by interventions that target the *Ae*. *aegypti* vector, also responsible for transmitting dengue, chikungunya, and yellow fever. For example, Costa Rica has reported over 20,000 dengue fever cases annually, and over 5,000 chikungunya cases since the disease appeared in 2014 [39].

If the $376 million proposed by the US President for foreign aid targeted at the Zika outbreak can avert infection among as little as 0.7% of the population of Latin America and the Caribbean, our analyses indicate that the intervention would be cost-effective. Averting this number of cases is highly feasible given that prior Zika outbreaks have exhibited attack rates ranging from 62% to 77% [15, 24].While vector control is the most immediately available tool for mitigating the Zika burden, the development of an efficacious vaccine would be a more sustainable long-term strategy. The budget requested by the US President to the Congress includes a provision of $200 million for research and development of a Zika virus vaccine. Since a successful vaccine could be used globally to prevent millions of Zika cases over many years, such an investment is more than justified.

Our tool identifies the combinations of price and effectiveness for which an intervention would be deemed cost-effective, but it does not predict the number of cases which an intervention will prevent, nor does it predict the net cost of an intervention. As mechanistic models describing Zika transmission and predicting the impact of interventions are developed, they can be integrated within the decision space delineated in this work, and by our web tool.

Our interactive application (http://zika.cidma.us/) provides a flexible tool for informing public health policy via a rigorous cost-benefit analysis of available options. While our examples focus on vector-control interventions, our framework would also be applicable to investments in vaccines or therapeutics. Difficult decisions related to next steps confront community members and leaders of areas that are currently facing, or will soon be facing, an epidemic of Zika. Given the potentially high health burden of Zika, the cost of inaction–or even insufficient action–may warrant significant expenditure.

## Supporting Information

S1 Table Parameters employed in the calculation of the probability of microcephaly given a Zika infection during the first trimester. (PDF)

S1 Fig Equations used to calculate the probability of microcephaly given a Zika infection of a mother during the first trimester of pregnancy. Symbols are defined in the S1 Table.
(PDF)

S2 Fig Cost of Zika-related microcephaly and GBS in Puerto Rico. Indirect costs (blue) are substantially higher than direct medical costs (red), and vary with the estimate of the probability of microcephaly given an infection during pregnancy and the probability of GBS given an infection (shaded regions) as well as with the attack rate (dotted vertical lines left to right: attack rate estimate of Chikungunya for Puerto Rico, attack rate estimate of Zika in French Polynesia and highest attack rate estimate of Zika for Yap Island).
(PDF)
