# Supplementary material for: A Cost-Effectiveness Tool for Informing Policies on Zika Virus Control
Source: PLoS Negl Trop Dis. 2016 May 20;10(5):e0004743. doi: 10.1371/journal.pntd.0004743 (PMC4874682; doi:10.1371/journal.pntd.0004743)
Supplement: S1 Table — (PDF) [file pntd.0004743.s001.pdf]

Symbol	Definition	Value	Source
P_M	Probability of microcephaly given Zika infection during the first trimester of pregnancy		M_Z/B_Z
M_Z	Zika-related microcephaly births in Northeast Brazil		$M - M_0$
M	Microcephaly births during outbreak in Northeast Brazil		sM_R
M_0	Microcephaly births not related to Zika during outbreak in Northeast Brazil		pB
M_R	Reported microcephaly births during outbreak in Northeast Brazil		$M_L + M_F$
M_C	Confirmed microcephaly births in Northeast Brazil		[17]
M_I	Investigated microcephaly births in Northeast Brazil		[17]
M_L	Microcephaly births reported thus far in Northeast Brazil		[17]
M_F	Forecast of microcephaly births for remainder of outbreak in Northeast Brazil		regression
B_Z	Births to mothers infected with Zika during period of risk in Northeast Brazil		aB
B	Births during outbreak in Northeast Brazil		$\frac{\ell}{365} b f_{\text{ne}} N$
a	Zika attack rate		[23, 24]
s	Sensitivity of microcephaly reporting		M_C/M_I
p	Microcephaly prevalence (per 10,000 births)		[18, 19]
ℓ	Duration of outbreak in Northeast Brazil (days)		regression
b	Brazilian birth rate		[22]
f_{ne}	Fraction of Brazilian population in Northeast Brazil		[20]
N	Brazilian population in 2015		[21]
