# Supplementary material for: A Cost-Effectiveness Tool for Informing Policies on Zika Virus Control
Source: PLoS Negl Trop Dis. 2016 May 20;10(5):e0004743. doi: 10.1371/journal.pntd.0004743 (PMC4874682; doi:10.1371/journal.pntd.0004743)
Supplement: S1 Fig — Symbols are defined in the S1 Table. (PDF) [file pntd.0004743.s002.pdf]

$$M_Z = M - M_0 \longleftarrow M_0 = p \times B$$

$$M = s \times M_R \longleftarrow M_R = M_L + M_F$$

$$P_M = \frac{M_Z}{B_Z} \qquad s = \frac{M_c}{M_I}$$

$$B_Z = a \times B \longleftarrow B = \frac{\ell}{365} b f_{\text{ne}} N$$
