# Supplementary figures and images for: A Cost-Effectiveness Tool for Informing Policies on Zika Virus Control
Source: PLoS Negl Trop Dis. 2016 May 20;10(5):e0004743. doi: 10.1371/journal.pntd.0004743 (PMC4874682; doi:10.1371/journal.pntd.0004743)

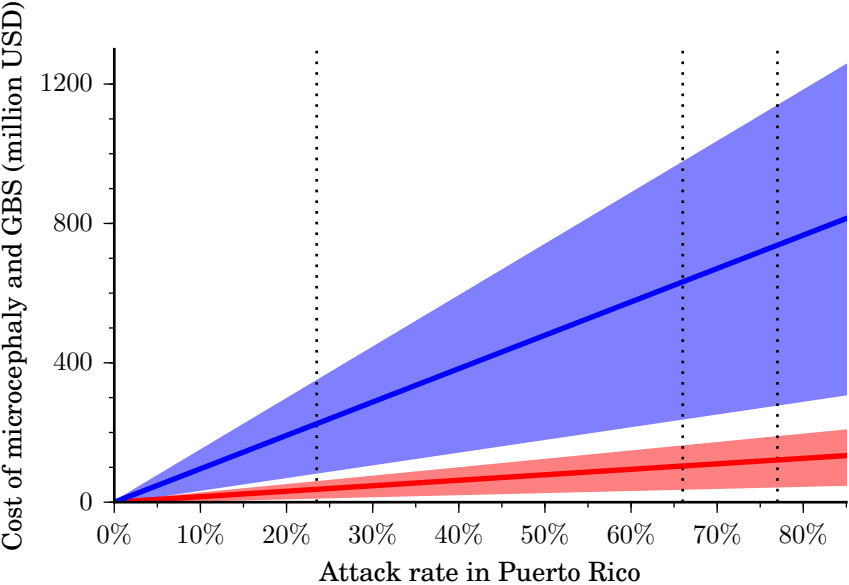

Supplement: S2 Fig — Indirect costs (blue) are substantially higher than direct medical costs (red), and vary with the estimate of the probability of microcephaly given an infection during pregnancy and the probability of GBS given an infection (shaded regions) as well as with the attack rate (dotted vertical lines left to right: attack rate estimate of Chikungunya for Puerto Rico, attack rate estimate of Zika in French Polynesia and highest attack rate estimate of Zika for Yap Island). (PDF) [file pntd.0004743.s003.pdf]
